# Treatment of Cystitis

**Published:** 1909-09

**Authors:** 


### TREATMENT OF CYSTITIS.

Dr. R. Ullmann, Chief of Staff of Bad Preblau, considers arhovin a good adjuvant in the treatment of cystitis (*Klin. Therap. Wochens.,* April 12, 1909). It combines antiseptic properties with analgesic and secretion-limiting effects. His results were so satisfactory that in some of the cystitis cases he was able to get along with the general dietetic and hygienic measures, without local treatment. In uncomplicated cystitis, arhovin quiets the vesical nerves and thus diminishes pain and urinary tenesmus; it lessens secretion; it clears the muco-purulent urine, at times in a few days; it increases the acidity of the urine and thus imparts to it bacterio-inhibitory properties, so that it cleanses the bladder and opposes the invasion of the inflammatory processes. The irritative symptoms and their causes, gonococci as well as other bacteria, are combated, so that complications are made rare. In obstinate, neglected cystitis, in prostatic cases and strictures, even with ammoniacal fermentation, the same effects were apparent.

In the complications of cystitis with inflammation of the renal pelvis, pyelonephritis, he was able to favorably influence the severe pains with arhovin. The suppuration in the renal pelvis abated and the diuresis was augmented.

He is thus in a position to conclude that arhovin is a most excellent adjuvant in the treatment of cystitis and its complications. It combines the virtues of the antiseptics with those of the balsams, without being detrimental to metabolism or the kidneys.

Dr. A. Kalmar, Vienna (*Therap. Ratgeber,* Feb. 20, 1909,) lays stress on the fact that astringent injections act mechanically by contracting the urethra and thereby expelling the cocci-containing secretion. But often the contractile waves proceed inwardly so that the cocci are carried up into the bladder, the kidneys, and the lymphatic passages. This occurs particularly when strong solutions are employed. When the cocci are imbedded in the deeper walls, the astringents can only do harm by rendering the conditions for the penetration of cocci more favorable. The author ascribes to this fact that strictures which often occur years after gonorrhea. On the other hand, arhovin-oil injections cause neither contraction of the tissues nor irritation; they are antiphlogistic, smoothing out and anesthetising the mucosæ. The oil is absorbed and thus acts deeply in the tissues, inhibiting the growth and migration of the cocci. Its use therefore is prophylactic of strictures as well as of joint rheumatism and endocarditis. He also favors the internal use of arhovin.

In two cases of condylomata acuminata the employment of arhovin ointment (3 parts arhovin with 50 parts each vaselin and lanolin) was promptly effective. Good results were also seen in fluor albus (arhovin internally and globule form). An especially interesting case of gonorrhea was that of a boy of 17, who neglected his symptoms till the urethra was highly inflamed, the orifice emitted a bloody secretion, the preputium was immovable, the glans was purulent, and the lymphatic glands were swollen. Under rest and vigorous employment of arhovin complete recovery was obtained in four weeks, without need of incising the bubos.

Dr. L. Szamek, Lantin Municipal Sanitarium in Baden (*Med. Blaetter,* February 6, 1909), concludes from his experience with arhovin that it is most useful in gonorrheal affections. It has great therapeutic value and exhibits not the least untoward effect, so that it may well be regarded as an excellent internal antigonorrheic.

